# Association between voriconazole exposure and Sequential Organ Failure Assessment (SOFA) score in critically ill patients

**DOI:** 10.1371/journal.pone.0260656

**Published:** 2021-11-24

**Authors:** Anne-Lise Bienvenu, Pierre Pradat, Alexandra Plesa, Vincent Leclerc, Vincent Piriou, Jean-Luc Fellahi, Laurent Argaud, Thomas Rimmelé, Jean Menotti, Frédéric Aubrun, Jean-Christophe Richard, Marie-Claude Gagnieu, François Parant, Christian Chidiac, Gilles Leboucher, Michel Tod, Sylvain Goutelle

**Affiliations:** 1 Service Pharmacie, Groupement Hospitalier Nord, Hospices Civils de Lyon, Lyon, France; 2 ICBMS CNRS 5246, Campus Lyon-Tech La Doua, Université de Lyon, Lyon, France; 3 Centre de Recherche Clinique, Groupement Hospitalier Nord, Hospices Civils de Lyon, Lyon, France; 4 Service d’anesthésie-réanimation, Groupement Hospitalier Sud, Hospices Civils de Lyon, Lyon, France; 5 Service d’anesthésie-réanimation, Groupement Hospitalier Est, Hospices Civils de Lyon, Lyon, France; 6 Service de réanimation médicale, Groupement Hospitalier Centre, Hospices Civils de Lyon, Lyon, France; 7 Service d’anesthésie-réanimation, Groupement Hospitalier Centre, Hospices Civils de Lyon, Lyon, France; 8 Service de Mycologie, Groupement Hospitalier Nord, Hospices Civils de Lyon, Lyon, France; 9 Service de réanimation chirurgicale, Groupement Hospitalier Nord, Hospices Civils de Lyon, Lyon, France; 10 Service de réanimation médicale, Groupement Hospitalier Nord, Hospices Civils de Lyon, Lyon, France; 11 Laboratoire de Biochimie et Biologie Moléculaire, UM Pharmaco-toxicologie Groupement Hospitalier Sud, Hospices Civils de Lyon, Lyon, France; 12 Comité des Anti-Infectieux, Hospices Civils de Lyon, Lyon, France; 13 Univ Lyon, Université Lyon 1, ISPB, Faculté de Pharmacie de Lyon, Lyon, France; 14 Univ Lyon, Université Lyon 1 UMR CNRS 5558, Laboratoire de Biométrie et Biologie Evolutive, Villeurbanne, France; Kaohsuing Medical University Hospital, TAIWAN

## Abstract

Therapeutic drug monitoring (TDM) is essential for voriconazole to ensure optimal drug exposure, mainly in critically ill patients for whom voriconazole demonstrated a large variability. The study aimed at describing factors associated with trough voriconazole concentrations in critically ill patients and evaluating the impact of voriconazole concentrations on adverse effects. A 2-year retrospective multicenter cohort study (NCT04502771) was conducted in six intensive care units. Adult patients who had at least one voriconazole TDM were included. Univariable and multivariable linear regression analyses were performed to identify predictors of voriconazole concentrations, and univariable logistic regression analysis, to study the relationship between voriconazole concentrations and adverse effects. During the 2-year study period, 70 patients were included. Optimal trough voriconazole concentrations were reported in 37 patients (52.8%), subtherapeutic in 20 (28.6%), and supratherapeutic in 13 (18.6%). Adverse effects were reported in six (8.6%) patients. SOFA score was identified as a factor associated with an increase in voriconazole concentration (p = 0.025), mainly in the group of patients who had SOFA score ≥ 10. Moreover, an increase in voriconazole concentration was shown to be a risk factor for occurrence of adverse effects (p = 0.011). In that respect, critically ill patients who received voriconazole treatment must benefit from a TDM, particularly if they have a SOFA score ≥ 10. Indeed, identifying patients who are overdosed will help to prevent voriconazole related adverse effects. This result is of utmost importance given the recognized COVID-19-associated pulmonary aspergillosis in ICU patients for whom voriconazole is among the recommended first-line treatment.

## Introduction

Therapeutic drug monitoring (TDM) is essential for certain drugs to ensure optimal drug exposure and avoid treatment failure, resistance, or toxicity [1]. This is especially true for drugs with wide inter- and intra-patient pharmacokinetic variabilities and drugs with a demonstrated correlation between drug plasma concentration and efficacy or toxicity. It is the case for voriconazole, a triazole antifungal, approved for the treatment of invasive aspergillosis (IA), oesophageal candidiasis, invasive candidiasis, scedosporiosis, and fusariosis [2, 3]. When used in critically ill patients, voriconazole demonstrated a large inter-patient variability in voriconazole plasma concentrations ranging from ≤ 1 mg/L for 37% of patients to > 5.5 mg/L for 19% of them [4]. Moreover, sub-therapeutic triazoles concentrations including voriconazole were linked to poor outcome compared with optimal concentrations [5, 6], whereas voriconazole supra-therapeutic concentrations were identified as a significant independent risk factor for hepatotoxicity in critically ill patients [7, 8]. Thus, voriconazole TDM is needed in critically ill patients to improve efficacy and safety as recently demonstrated [9].

In that respect, Infectious Disease Society of America (IDSA) recommends TDM for IA once the steady state has been reached for patients receiving triazole-based therapy or other therapies for which drug interactions with azoles are anticipated (strong recommendation; moderate-quality evidence) [10]. Besides, panel members of scientific societies including European Society of Intensive Care Medicine (ESICM) [11] recommends voriconazole TDM to be routinely performed when it is used in critically ill patients; indeed, TDM-guided voriconazole dosing improved clinical response and reduced voriconazole discontinuation due to adverse events [12]. Finally, timely TDM for triazole antifungal agents is considered by the Mycoses Study Group Education and Research Consortium as a core element for best practices in antifungal stewardship [13].

In this context, we conducted a retrospective multicenter cohort study in Lyon University Hospital based on the determination of serum voriconazole trough concentrations in adult patients who received voriconazole as a treatment during their ICU stay. The main objective of this study was to describe factors associated with voriconazole trough concentrations on the first TDM occasion. The secondary objectives were to evaluate the impact of trough voriconazole concentrations on occurrence of adverse effects.

## Materials and methods

### Study design

This study was a 2-year (2018–2019) retrospective multicenter cohort study using hospital database. Six intensive care units (ICUs) including medical (two units), surgical (one unit), and mixed medical and surgical (three units) ICUs, located in four medical centers were involved. Adult patients > 18 years-old who received voriconazole as a treatment and had at least one TDM during ICU stay were included in the study. There was no upper age limit for inclusion. Patients who were not sampled 12 +/- 2 h after voriconazole administration, patients who received an antifungal combination, pediatric patients, and patients who were not admitted to an ICU, were not included. Serum voriconazole concentrations were determined in the pharmacology laboratory of Lyon hospital using a validated liquid chromatography/tandem mass spectrometry (LC/MS/MS) method. The lower limit of quantification was 0.05 mg/L and the inter-day precision was less than 5.9% over the calibration range (0.05–5.89 mg/L). Voriconazole trough concentration (Cmin) was sampled 12 +/- 2 h after voriconazole administration. Target trough voriconazole serum concentration ranged from 1 to 5 mg/L [11]. According to this target and based on results observed on the first TDM occasion, patients were categorized into three groups: patients who had optimal voriconazole concentrations (Cmin = 1 to 5 mg/L), supratherapeutic concentrations (Cmin > 5 mg/L) and subtherapeutic concentrations (Cmin < 1 mg/L). During the study period, major diagnostic, therapeutic, and infection control standards, remained unchanged. The study was registered in Clinicaltrials.gov (NCT04502771). This study was part of an antifungal stewardship program implemented in our institution which major aim is to promote the optimal use of antifungals.

### Ethics statement

This was a non-interventional study, without any additional procedure. Data were collected during routine patient care. This study was conducted in accordance with the Declaration of Helsinki and national and institutional standards. The study was approved by the institutional Ethics Committee (N°20–11). Due to the retrospective nature of the study, formal consent was not required. Electronic records were under the auspice of the French National Committee for Data Protection and Freedom of Information.

### Data collection

The following data were collected using hospital database: age, weight on the day of TDM, gender, main underlying disease, Glasgow coma score on ICU admission, Sequential Organ Failure Assessment (SOFA) score on the day of TDM, septic shock (identified by vasopressor requirement) on the day of TDM using the worst parameters measured during the prior 24 hours, use of mechanical ventilation, biological parameters (including C-reactive protein, procalcitonin, white blood cells count, platelets count, total proteins, albumin, glomerular filtration rate, serum-glutamyl-oxaloacetate-transferase (SGOT), serum-glutamyl-pyruvate-transaminase (SGPT), gamma-glutamyl-transferase (GGT), conjugated bilirubin, total bilirubin), voriconazole dosing regimen, co-administration of cytochrome P450 inhibitor and/or inducer (including CYP3A4, CYP2C9, CYP2C19) according to voriconazole summary of product characteristics, trough voriconazole serum concentration, mycological diagnosis, and occurrence of adverse effects at the end of antifungal treatment. SOFA Score is a mortality prediction score that is based on the degree of dysfunction of six organ systems including respiratory, cardiovascular, hepatic, coagulation, renal, and neurological systems. When calculated using the worst parameters measured during the prior 24 hours, it provides a stratification of the mortality risk in ICU patients. To ensure reproducibility and completeness of data extraction, an Excel spread sheet (Microsoft Corp., Redmond, WA, USA) compiling all variables to be extracted was used. Pharmacists were in charge of data collection. Data extraction was double-checked. Disagreements over data extraction were resolved by discussion. Provided data were centrally checked for completeness, plausibility, and integrity before synthesis. The checklist of the Strengthening the Reporting of Observational Studies in Epidemiology (STROBE) Statement hosted by the Enhancing the QUAlity and Transparency Of health Research (EQUATOR) network was used as a methodological support.

### Evaluation of adverse effects

Adverse events, if available, were collected in each patient’s chart. According to the Guideline on good pharmacovigilance practices of European Medicines Agency (EMA), an adverse effect is a response to a medicinal product which is noxious and unintended [DIR 2001/83/EC Art 1(11)]. Response in this context means that a causal relationship between a medicinal product and an adverse event is at least a reasonable possibility. Hepatotoxicity was defined as a hepatic injury revealed by a significant elevation of SGOT and/or SGPT (≥ 2 fold) during voriconazole treatment. Neurotoxicity was defined as an alteration of the normal activity of the nervous system and was revealed by hallucinations occurring during voriconazole treatment.

### Statistical analysis

Categorical variables were presented with numbers and percentages whereas continuous variables were presented with means +/- standard deviation (SD). For categorical variables, comparisons between groups were performed using the Chi-squared test or the Fisher’s exact test, as appropriate. For continuous variables, comparisons between groups were performed by a t-test in case of normal distribution and sample size >30 in each group or by the non-parametric Mann-Whitney U test otherwise.

The statistical analysis was designed according to the sample size (n = 70). To identify independent predictors of voriconazole concentrations, univariable and multivariable linear regression analyses were performed. In the multivariable analysis, all variables with a p-value<0.1 in univariable analysis and variables known or highly suspected to be associated with the outcome were included. To avoid collinearity that reduces the precision of the estimated coefficients, variables contributing to multicollinearity (i.e. vasopressors, septic shock, mechanical ventilation, voriconazole daily dose) were excluded from the multivariable analysis. Indeed, vasopressors, septic shock, and mechanical ventilation, are clinically correlated with SOFA score, and voriconazole daily dose with voriconazole trough concentration. Beside, Akaike Information Criterion, an estimator of prediction error, was used to select the best statistical model for the multivariable analysis. Method used for handling missing data was case deletion considering that the assumption of missing completely at random was satisfied. Estimates with their respective 95% confidence intervals (CI) are presented. A univariable logistic regression analysis was used to identify the potential relationship between voriconazole concentrations and occurrence of adverse effects. Odds ratios (OR) and corresponding 95% CIs are shown.

R-4.0.2 software (R Foundation for Statistical Computing, Vienna, Austria) was used for descriptive analysis, as well as for univariable and multivariable analyses. P-value < 0.05 was considered statistically significant.

## Results

### Baseline characteristics

**Fig 1:**
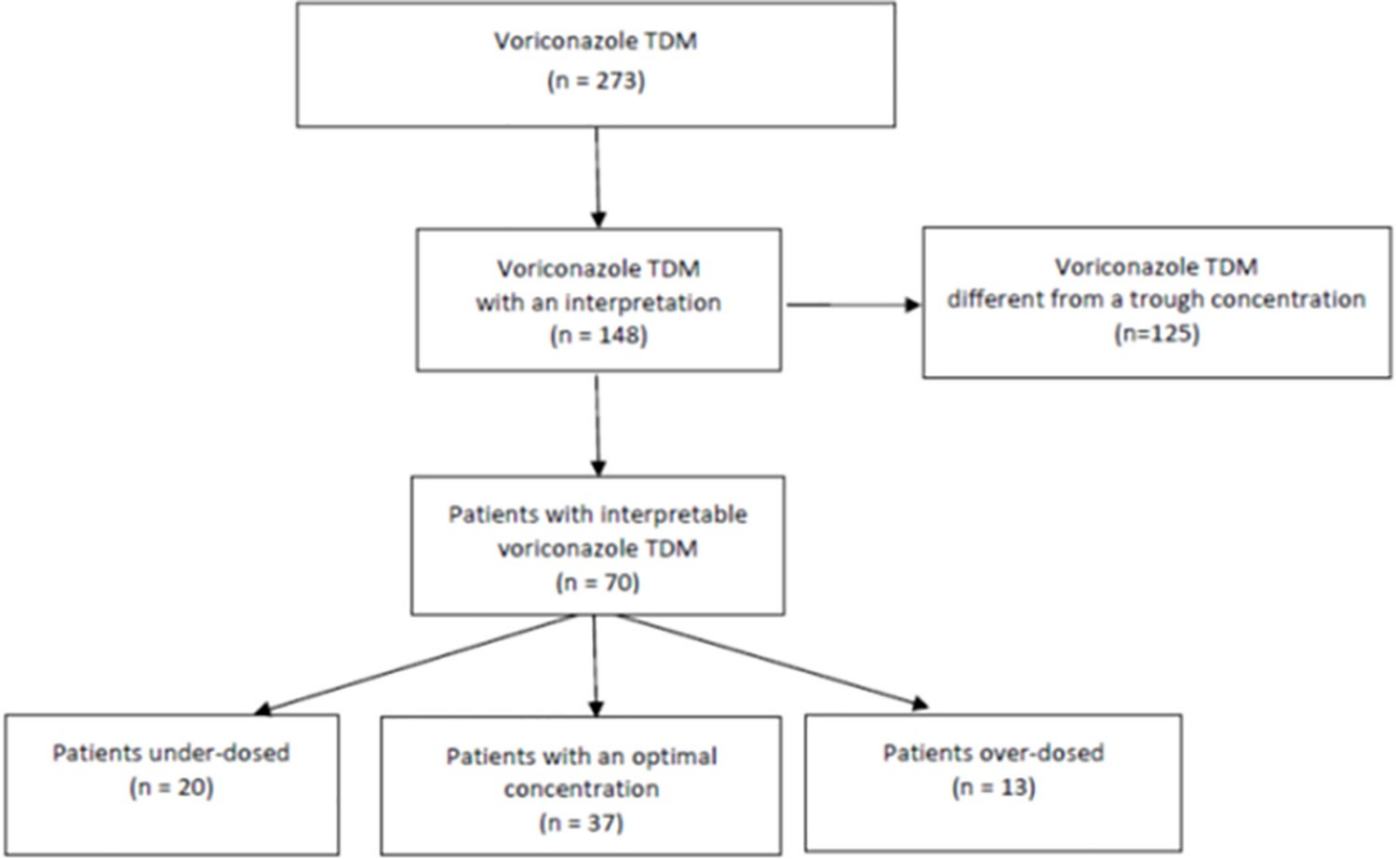
Study flow chart.

During the 2-year study period, 273 voriconazole concentrations were collected (Fig 1). One hundred and forty-eight (n = 148) of them met the definition of voriconazole trough concentrations and 125 were discarded as they did not match the definition of voriconazole trough concentration. Among the eligible voriconazole trough concentrations (n = 148), 70 concentrations sampled on the first TDM occasion were included in the analysis. The study population consisted of 70 patients (46 male and 24 female) aged 56,3 +/- 14,4 years, amongst whom 32 patients had only one TDM occasion and 38 had more than one TDM occasion. Baseline characteristics of the 70 included patients on the first TDM occasion are detailed in Table 1. Thirty-one (n = 31; 44.3%) patients had a haematological malignancy, 11 (15.7%) had a solid organ transplantation, six (8.6%) a solid cancer, six (8.6%) an acute respiratory distress syndrome, and 16 (22.8%) another underlying disease. Most of them (n = 46) received intravenous voriconazole and 24, oral voriconazole. Thirty-four (n = 34; 48.6%) patients had a daily dose below or equal to 400 mg daily and 36 (51.4%), a daily dose over 400 mg. Among the included patients who had a mycological diagnosis (n = 39), 24 (61.5%) received voriconazole for an invasive aspergillosis, five (12.8%) for an invasive candidiasis, and ten (25.7%) for another invasive fungal disease. On the first TDM occasion, optimal voriconazole concentrations were reported in 37 patients (52.8%), subtherapeutic concentrations in 20 patients (28.6%), and supratherapeutic concentrations in 13 patients (18.6%). Adverse effects (hepatotoxicity (n = 4), neurotoxicity (n = 2)) associated with voriconazole treatment were reported in six (8.6%) patients (Table 1).

**Table 1:** Baseline characteristics of critically ill patients on the first voriconazole therapeutic drug monitoring. Data presented are means +/- standard deviations or numbers and corresponding percentages (%).

Characteristics	Patients (n = 70)
**Clinical features**	
Sex, M/F	46/24
Age (years), Mean+/-SD	56.3 +/- 14.4
Weight (kg), Mean+/-SD	72.2 +/- 17.2
Underlying disease	
Haematological malignancies, n (%)	31 (44.3)
Solid organ transplant, n (%)	11 (15.7)
Cancer, n (%)	6 (8.6)
Acute respiratory distress syndrome, n (%)	6 (8.6)
Others, n (%)	16 (22.8)
Septic shock, n (%)	41 (58.6)
SOFA (n = 59), Mean+/-SD	7.7 +/- 4.6
Glasgow score (n = 65), Median [IQR25-IQR75]	13 [8–15]
Vasopressors, n (%)	31 (44.3)
Mechanical ventilation, n (%)	41 (58.6)
**Biological parameters**	
C reactive protein (mg/L) (n = 34), Mean+/-SD	155.5 +/- 112.9
Procalcitonin (ng/mL) (n = 20), Mean+/-SD	6.5 +/- 16.0
White blood cells (G/L), Mean+/-SD	10.4 +/- 9.7
Platelets (G/L), Mean+/-SD	156.1 +/- 147.0
Proteins (g/L), Mean+/-SD	53.2 +/- 10.8
Albumin (g/L) (n = 50), Mean+/-SD	21.0 +/- 4.8
Glomerular filtration rate (mL/min) > 90 mL/min/1.73m^2^, n (%)	38 (54.3)
SGOT (U/L) (n = 69), Mean+/-SD	103.2 +/- 254.7
SGPT (U/L) (n = 69), Mean+/-SD	83.0 +/- 191.5
GGT (U/L) (n = 69), Mean+/-SD	179.1 +/- 195.8
Conjugated bilirubin (μmol/L) (n = 35), Mean+/-SD	54.6 +/- 69.6
Total bilirubin (μmol/L) (n = 66), Mean+/-SD	43.6 +/- 73.3
**Treatments**	
Concomitant cytochrome P450 inhibitor, n (%)	25 (35.7)
Concomitant cytochrome P450 inducer, n (%)	3 (4.3)
Voriconazole daily dose (mg/day), Mean+/-SD	517 +/- 171
Intravenous route	46 (65.7)
Oral route	24 (34.3)
Voriconazole trough concentration (n = 70), Mean+/-SD	3.0 +/- 2.5
Exposure	
Within therapeutic range (1–5 mg/L), n (%)	37 (52.9)
Underexposure (< 1 mg/L), n (%)	20 (28.6)
Overexposure (> 5 mg/L), n (%)	13 (18.6)
Mycological diagnosis (n = 39)	
Aspergillosis, n (%)	24 (61.5)
Candidiasis, n (%)	5 (12.8)
Invasive fungal disease, n (%)	10 (25.7)
**Outcome**	
Adverse effects, n (%)	6 (8.6)

GGT: gamma-glutamyl- transferase; SD: standard deviation; SGOT: serum-glutamyl-oxaloacetate-transferase; SGPT: serum-glutamyl-pyruvate-transaminase; SOFA: Sequential Organ Failure Assessment.

### Factors associated with voriconazole concentration

**Fig 2:**
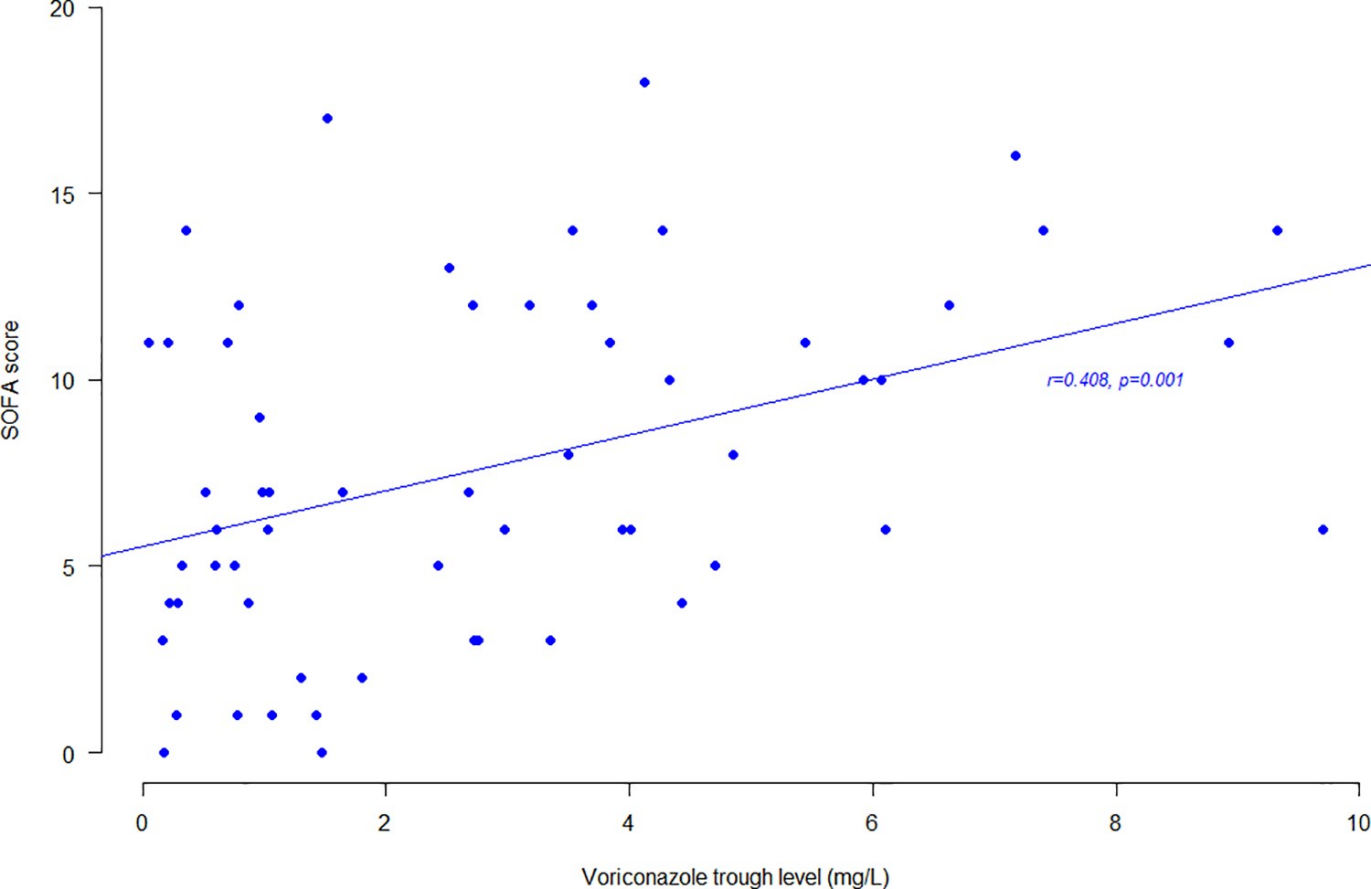
Correlation between SOFA score and voriconazole trough levels. The dots represent the individual pairs for the X–Y variables. The straight line is the linear regression line.

Variables associated with voriconazole concentration are shown in Table 2. According to univariable linear regression analysis, weight, septic shock, SOFA score, use of vasopressors, mechanical ventilation, and daily dose, were significantly associated with an increase in voriconazole concentration. Interestingly, voriconazole concentrations were significantly higher for critically ill patients with SOFA score ≥ 10 compared to SOFA score < 10 (4.0 +/- 2.8 mg/L and 2.1 +/- 2.0 mg/L, respectively (p = 0.009)), whereas voriconazole daily doses were not significantly different among those groups (p>0.1). Besides, occurrence of underdosing < 1 mg/L was significantly more frequent in patients receiving a daily dose below 400 mg compared to a daily dose over 400 mg (41.2% (14/34) vs 16.6% (6/36), respectively (p = 0.023)), whereas occurrence of overdosing > 5 mg/L was significantly more frequent in patients receiving a daily dose over 400 mg compared to a daily dose below 400 mg (30.6% (11/36) vs 5.9% (2/34), respectively (p = 0.012)). According to multivariable linear regression analysis using the best model according to Akaike Information Criterion (i.e sex, weight, SOFA score, use of CYP450 inhibitor) and excluding factors of multicollinearity (i.e. vasopressors, septic shock, mechanical ventilation, voriconazole daily dose), SOFA score was confirmed to be a significant factor associated with an increase in voriconazole concentration (p = 0.025): a one-point increase in SOFA score was associated with a 0.19 mg/L increase in voriconazole concentration. Fig 2 illustrates the correlation between SOFA score and voriconazole trough levels (p = 0.001).

**Table 2:** Univariable and multivariable analyses of variables potentially associated with voriconazole trough concentration. Data presented are β coefficients (95% CI) and their corresponding *P* values.

Characteristics	Univariable analysis		Multivariable analysis	
	β coefficient (95% CI)	*P* value	β coefficient (95% CI)	*P* value
**Clinical features**				
Sex, M vs F	1.1 (-0.12–2.3)	0.075	0.90 (-0.53–2.3)	0.214
Age (years)	0.03 (-0.01–0.07)	0.122		
Weight (kg)	0.04 (0.01–0.08)	0.013	0.01 (-0.04–0.05)	0.824
Septic shock	1.5 (0.33–2.6)	0.012		
SOFA (n = 59)	0.22 (0.09–0.35)	0.001	0.19 (0.02–0.36)	0.025
Glasgow score (n = 65)	-0.11 (-0.25–0.03)	0.121		
Vasopressors	1.8 (0.66–2.9)	0.002		
Mechanical ventilation	1.2 (0.02–2.4)	0.047		
**Biological parameters**				
C reactive protein (n = 34)	0.00 (-0.01–0.01)	> 0.9		
Procalcitonin (n = 20)	0.03 (-0.02–0.09)	0.236		
White blood cells	0.01 (-0.05–0.07)	0.730		
Platelets	0.00 (0.00–0.00)	> 0.9		
Proteins	-0.03 (-0.09–0.03)	0.285		
Albumin (n = 50)	-0.03 (-0.18–0.13)	0.750		
Glomerular filtration rate > 90 mL/min/1.73m^2^	-0.10 (-1.3–1.1)	0.874		
SGOT (n = 69)	0.00 (0.00–0.00)	> 0.9		
SGPT (n = 69)	0.00 (0.00–0.00)	0.489		
GGT (n = 69)	0.00 (0.00–0.01)	0.621		
Conjugated bilirubin (n = 35)	0.00 (-0.01–0.02)	0.789		
Total bilirubin (n = 66)	0.00 (0.00–0.01)	0.274		
**Treatments**				
Concomitant cytochrome P450 inhibitor	1.1 (-0.14–2.3)	0.082	1.0 (-0.29–2.3)	0.126
Concomitant cytochrome P450 inducer	-2.0 (-4.9–0.88)	0.168		
Voriconazole daily dose (mg)	0.01 (0.00–0.01)	< 0.001		
Oral route	-0.86 (-2.1–0.38)	0.172		

GGT: gamma-glutamyl- transferase; SD: standard deviation; SGOT: serum-glutamyl-oxaloacetate-transferase; SGPT: serum-glutamyl-pyruvate-transaminase; SOFA: Sequential Organ Failure Assessment.

### Outcome associated with voriconazole concentration

**Fig 3:**
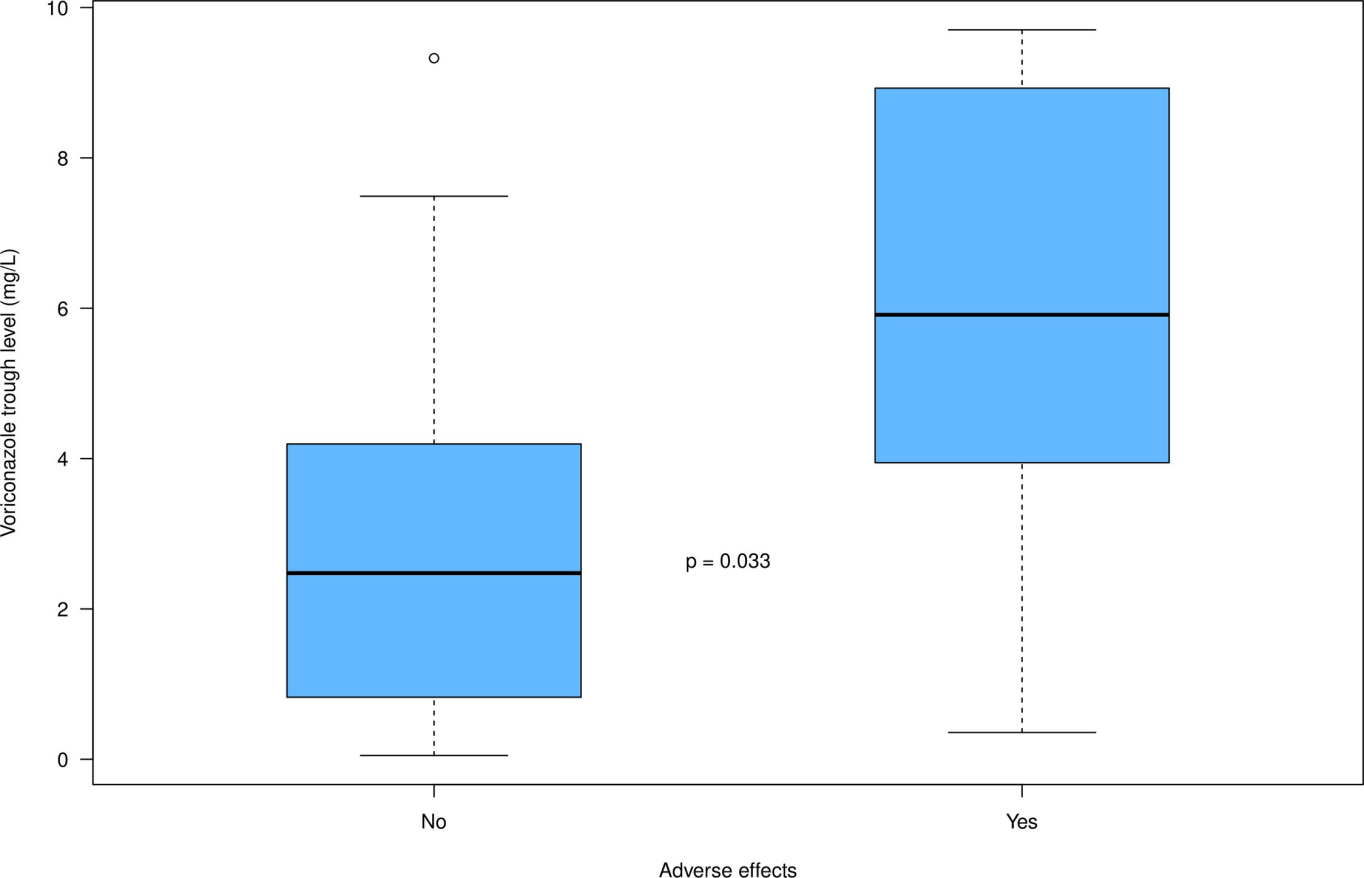
Box plots of voriconazole trough levels according to the presence or absence of adverse effects. For each box of the box plots, the centre line represents the median, the bottom line represents the 25th percentiles and the top line represents the 75th percentiles. The whiskers of the box plots show 1.5 interquartile range (IQR) below the 25th percentiles and 1.5 IQR above the 75th percentiles, and outliers are represented by small circles.

According to univariable logistic regression analysis, an increase in voriconazole concentration was associated with occurrence of adverse effects (OR (95% CI) = 1.56 (1.11–2.20); p = 0.011): a 1 mg/L increase in voriconazole concentration was associated with a 56% increased risk of adverse effects. Indeed, voriconazole concentrations were found to be significantly higher for patients who experienced adverse effects attributed to voriconazole compared to patients who did not (5.8 +/- 3.4 mg/L and 2.7 +/- 2.2 mg/, respectively (p = 0.03)). Fig 3 displays box plots that illustrates the association between voriconazole trough levels and adverse effects (p = 0.033).

## Discussion

This 2-year retrospective multicenter cohort study aimed at identifying factors associated with voriconazole concentrations on the first TDM occasion. The need for voriconazole TDM studies in critically ill patients was demonstrated as claimed in the Intensive care medicine research agenda on invasive fungal infection in critically ill patients [1]. Indeed, on the first TDM occasion, optimal trough voriconazole concentrations were reported in only half of the patients (52.8%), and this, despite a daily dose over 400 mg in most of the patients. Most of critically ill patients who benefit from a voriconazole TDM suffered from a haematological disease, thus requiring a rapid and optimal exposure to voriconazole for an efficient treatment of their invasive fungal infections. The same challenge is faced in critically ill COVID-19 patients who developed COVID-19 associated pulmonary aspergillosis (CAPA) treated with voriconazole as a first-line treatment [14]. Considering CAPA as an additional contributing factor to mortality [15], an optimal exposure to voriconazole in those critically ill patients is urgently needed.

According to multivariable analysis, a significant association between SOFA score and voriconazole concentrations was demonstrated. SOFA score is a mortality prediction score used in ICU patients including septic patients. It is based on the degree of dysfunction of six organ systems including respiratory, cardiovascular, hepatic, coagulation, renal, and neurological systems. Among organ dysfunction, liver impairment may occur in patients with high SOFA scores. The liver is indeed a target for sepsis-related injury, including hypoxic hepatitis due to ischemia and cholestasis due to altered bile metabolism [16]. As voriconazole is metabolised by the hepatic cytochrome P450 isoenzymes and eliminated via hepatic metabolism, voriconazole metabolism is altered in case of liver impairment. Consequently, voriconazole overexposure may occur in patients with liver impairment. Among our patients with high SOFA scores (≥10), 95% of them were septic and 70% of them had a liver impairment according to the high levels of their liver enzymes (SGOT, SGPT, and/or GGT). Then, liver impairment deplored in this subgroup of patients led to a reduction in voriconazole hepatic metabolism explaining in part voriconazole overexposure in patients with high SOFA scores.

In contrast to what had been previously reported in critically ill patients, age, sex, serum albumin concentration, or non-intravenous administration, were not found in this study to be associated with subtherapeutic voriconazole concentrations [9], and bilirubin, CRP, or intravenous treatment, to be associated with supratherapeutic concentrations [7, 9]. Importantly, conjugated bilirubin and CRP were collected in only half of patients in our study making the analysis uncertain for these two biological parameters.

Few adverse effects attributed to voriconazole treatment were reported in patients’ charts (8.6% of patients), then suggesting acceptable tolerance of voriconazole. Nevertheless, an univariable logistic regression analysis demonstrated that a 1 mg/L increase in voriconazole concentration was responsible for a 56% increased risk of voriconazole related adverse effects including hepatotoxicity and neurotoxicity, as previously demonstrated [8, 17].

This study has some limitations. First, it was a retrospective study that did not allow to control time of blood sampling for voriconazole TDM which explained the low number of patients (n = 70) included during a 2-year period. For some variables including adverse effects, a low number of events (n = 6) was reported thus potentially affecting the stability of the logistic model. Second, the first voriconazole TDM was used to categorize patients into groups: in fact, those groups were used for the qualitative analysis, but not for the univariable and multivariable analyses for which the predictor variable was voriconazole concentration. Third, mycological follow-up was available for 15 patients only, thus did not allow to explore this variable further.

## Conclusions

First, this study demonstrated that an increase in SOFA score was associated with an increase in voriconazole trough concentration in critically ill patients. Second, an increase in voriconazole trough concentration was related with a higher risk of voriconazole related adverse effects. Therefore, avoiding voriconazole overdosing in critically ill patients, particularly if they have a SOFA score ≥ 10, will help to prevent voriconazole related adverse effects. This result is of utmost importance given the recognized COVID-19-associated pulmonary aspergillosis in ICU patients for whom voriconazole is among the recommended first-line treatment [14]. As previously demonstrated [18], antifungal stewardship activities would help to promote voriconazole optimal exposure and control adverse effects.
